# The Immediate Effects of Vibrotexture Shoe Insoles on Quiet Standing Balance and Lower Limb Muscle Activity in Healthy Young Adults

**DOI:** 10.1002/jfa2.70150

**Published:** 2026-03-19

**Authors:** Megan Trotman, Avni Hurkat, Kylie Tucker, Thomas Cattagni, Anna L. Hatton

**Affiliations:** ^1^ School of Health and Rehabilitation Sciences The University of Queensland Brisbane Australia; ^2^ School of Biomedical Sciences The University of Queensland Brisbane Australia; ^3^ Movement, Interactions, Performance Nantes Université MIP Nantes France

**Keywords:** balance, electromyography, feet, insoles, postural control, sensory feedback, stimulation

## Abstract

**Introduction:**

Shoe insoles that provide a single source of plantar sensory stimulus, such as texture or vibration, can improve measures of standing balance. Vibrotexture insoles, designed to provide multiple sources of sensory stimuli, may provide greater benefits. This study aimed to investigate the immediate effects of wearing Vibrotexture insoles on quiet standing balance and lower limb muscle activity in healthy young adults.

**Methods:**

Thirty healthy young adults (16 males, 23.6 ± 3.9 years) performed standing balance tests (eyes open/closed, firm/foam surface) wearing four different insoles (vibrotexture, textured, vibrating, and control) within standardized shoes. Balance outcomes included center of pressure (COP) velocity, COP anteroposterior and mediolateral (ML) path length and range. Lower limb muscle amplitude was examined using electromyography at the medial gastrocnemius, soleus, peroneus longus, rectus femoris, biceps femoris, and gluteus medius (dominant leg). Perceived insole comfort and user experience was reported. One‐way Friedman tests were used to compare differences between insoles.

**Results:**

For the primary aim of interest, there were no differences in balance measures or muscle activity between the vibrotexture insoles and other insole conditions. Exploratory secondary analyses revealed that COP ML path length was less while wearing the textured compared to vibrating insoles (eyes open, firm surface) (*p* = 0.022), and rectus femoris muscle amplitude was less while wearing the textured compared to control insoles (eyes open, foam surface) (*p* = 0.027). The vibrotexture insoles were perceived as less comfortable than the control insoles (*p* < 0.013). Participants commonly reported the sensory‐stimulating insoles to feel “spiky”, “tingly”, or “rough”.

**Conclusion:**

Wearing vibrotexture insoles for the first time does not appear to alter standing balance in young adults. However, textured insoles do alter COP ML path length and rectus femoris amplitude. These findings highlight the need to investigate vibrotexture insoles in more dynamic tasks and in populations with reduced foot sensation, where their effects may be more pronounced.

## 1 Introduction

Maintaining upright standing balance involves the processing and integration of sensory information from vestibular, visual, and somatosensory pathways in the central nervous system to inform an appropriate motor response [1]. Emerging strategies to augment sensory information for postural control include footwear devices (e.g., shoe inserts, orthoses) that are designed to stimulate cutaneous receptors on the plantar surface of the feet [2, 3, 4]. Sensory‐stimulating footwear devices include insoles that comprise textured (raised geometric shapes) or vibrating elements.

For healthy young adults, wearing textured insoles or standing on a textured floor surface has shown immediate changes in quiet standing balance, including reduced center of pressure (COP) displacement, path length, and peak velocity, when compared to a control condition (e.g., smooth insoles or floor surfaces) [5, 6, 7, 8]. Further, insoles that deliver vibratory stimuli can lead to reductions in COP displacement, elliptical area, and velocity during quiet standing in healthy young adults, compared to control (no vibration) insoles [4, 9, 10]. However, some studies have reported no effects of textured floor surfaces [11, 12], and vibrating insoles [13] on COP measures in healthy young adults. These discrepancies may indicate that the effectiveness of sensory‐stimulating insoles depends on factors such as insole or surface design, or the sensory demands of the balance task.

Possible motor control strategies that underlie the effects of sensory‐stimulating insoles on postural control measures remain unclear. Changes in lower limb muscle activity may be one possible mechanism of action: yet a limited number of studies [9, 11, 12] have explored whether wearing textured or vibrating insoles alter muscle activity during standing balance in healthy young adults. One study [9] examined the effects of vibrating insoles on lower limb muscle activity during quiet stance, and reported reduced tibialis anterior, medial gastrocnemius, and lateral gastrocnemius muscle amplitude compared to control insoles, in healthy young adults. However, standing on textured surfaces does not appear to alter lower limb muscle activity during balance tasks in healthy young adults relative to control conditions [11, 12]. Given the small body of prior work, the current study aimed to further our understanding of the neuromuscular processes by which sensory‐stimulating insoles may alter balance control.

To date, research has focused on the investigation of insoles with a single source of sensory stimulus (texture or vibration), with evidence showing improvements in balance control in healthy young adults. However, no studies have examined the effects of insoles that provide multiple sources of sensory stimuli. We propose novel vibrotexture insoles, combining both vibration and texture. Vibrotexture insoles have the potential to activate a larger range (i.e., different types) of plantar cutaneous mechanoreceptors and may bring about greater benefits to balance control, relative to single‐sensory stimuli insole design. This study examined balance and muscle activity in younger adults to establish baseline effects of vibrotexture insoles in a healthy population without age‐related decline, and to enable direct comparison with prior work on single‐stimulus insole effects during quiet standing. We manipulated the sensory environment across four stance conditions (eyes open/closed; firm/foam surface) to create more challenging postural demands, thereby increasing the potential to detect insole‐related effects through greater reliance on enhanced plantar surface stimulation [14].

Therefore, the aim of this study was to explore the immediate effects of wearing vibrotexture insoles on measures of standing balance (peak COP path length, velocity, and range) and lower limb muscle activity (amplitude), in healthy young adults, compared to wearing textured insoles, vibrating insoles, and control insoles. We hypothesized that wearing vibrotexture insoles would reduce COP path length, velocity, and range, and amplitude of lower limb muscle activity, compared to the other insoles.

## 2 Materials and Methods

### 2.1 Participants

Thirty healthy young adults (16 males) were recruited through advertisements to staff and students at The University of Queensland, Brisbane, Australia. All data collection took place in the Gait Laboratory at The University of Queensland, during a single laboratory assessment that was approximately 2 h duration. Participants were included if they were aged 18–30 years, were able to stand unsupported without use of an assistive device (e.g., cane), and were willing to wear insoles during balance tasks. Exclusion criteria were a self‐reported history of neurological disease, peripheral neuropathy, vestibular conditions, or lower limb pain or injury in the past 12 months that limited activity. Participants provided verbal and written informed consent prior to enrollment. The study was approved by The University of Queensland Human Research Ethics Committee (#HE002481).

### 2.2 Insoles

**FIGURE 1:**
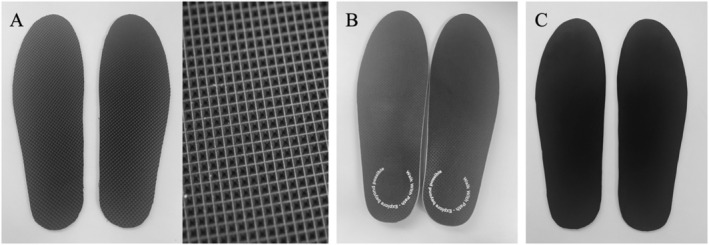
(A) Textured insoles, with geometric pattern of top surface, (B) vibrating insoles, and (C) control insoles.

Participants wore four different insoles in a random, counterbalanced order: textured insoles, vibrating insoles, vibrotexture insoles (texture and vibration), and control insoles (no texture or vibration). The textured insoles (Figure 1A) comprised raised pyramidal peaks (with 2.5 mm peak‐to‐peak distances) across the top surface (Evalite Pyramid Lightweight EVA Soling [A50], 3 mm thick, black, OG 1549; Algeos PTY Ltd., Dandenong South, Australia). The vibrating insoles (Figure 1B) (Walk With Path, London/Copenhagen) were constructed from a thermoplastic elastomer, Poron, microfiber lining, and included foot arch contouring. The insoles delivered intermittent perceptible vibration (operated using a mobile app) under the heel and forefoot that was synchronized with the direction of sway (heel motor activation during posterior sway, forefoot motor activation during anterior sway). The control insoles (Figure 1C) had a smooth, flat surface (Aortha Medium Density EVA‐340 [A50], 3 mm thick, black, OG1304; Algeos PTY Ltd., Dandenong South, Australia). The insole thickness and foot arch contouring was consistent across all four insole conditions. Participants wore thin disposable socks (Graaf Imports, Australia), and the insoles were inserted into standardized shoes (Volley International Canvas, China) available in a range of unisex shoe sizes. The insoles were available in three different sizes, selected based on the participant's shoe size. Prior to testing each insole, participants walked for 5 min while wearing the insoles and shoes to allow for familiarization.

### 2.3 Procedures

#### 2.3.1 Standing Balance

Balance data were collected using a Kistler force platform (9260AA6, Winterthur, Switzerland) and sampled at a rate of 1000 Hz. Participants maintained a standardized foot position (heels placed apart at 1/10th participant's height, angled at 14°) [15], with their arms hanging by their sides [16]. Standing balance was assessed over two trials of 30 s for four randomized balance tests: eyes open and eyes closed, on a firm surface and foam surface (foam block 500 × 500 × 150 mm; density 31 kg/m^3^; hardness 320 N; Dyman Foams, Geebung, Australia). While standing with eyes open, participants focused on a square visual target (7.5 × 7.5 cm) fixed onto a wall directly ahead (3 m from the center of the force platform), adjusted to eye level. All balance tests were performed whilst wearing one insole before changing to the next insole condition.

#### 2.3.2 Muscle Activity

Surface electromyography (EMG) recordings were collected from the gluteus medius, rectus femoris, biceps femoris, peroneus longus, medial gastrocnemius, and soleus, of the dominant leg (determined by asking participants which leg they typically use to kick a ball) [16]. Prior to electrode placement, participant's skin was cleaned with an alcohol swab, and shaved if hair was covering the electrode site, to minimize skin‐electrode impedance. Surface EMG electrodes (Trigno, Delsys, LA, USA) were placed over the muscle belly, in the direction of the muscle fibers, according to SENIAM recommendations [17]. EMG signals were recorded at a sampling frequency of 1000 Hz.

#### 2.3.3 Foot Sensation

To characterize the sample, foot sensory function was measured bilaterally. Semmes–Weinstein monofilaments were used to assess light‐touch pressure sense at the first and fifth metatarsal heads, heel, plantar surface of the distal hallux, and dorsal aspect of the midfoot [18]. Vibration perception was assessed using a neurothesiometer (amplitude 0–50V) (Horwell, Scientific Laboratory Supplies, Nottingham, U K) at the plantar surface of the distal hallux and first metatarsal head. Proprioception was assessed using the ankle joint position sense test [19]. The difference (error in degrees) between the target angle and repositioned angle was measured using an internet‐based goniometer (average of three trials per foot) [20].

#### 2.3.4 Participant Reported Outcomes

Participants were asked to complete a series of questionnaires concerning their basic demographics (e.g., sex, height), physical activity levels (The Active Australia Survey [21]), balance confidence (Activities‐Specific Balance Confidence Scale [22]), and foot health specific quality of life (Foot Health Status Questionnaire [23]). Participants were asked to rate their perceived level of insole comfort using a 100 mm visual analog scale (0 = extremely uncomfortable and 100 = extremely comfortable) [24] after wearing the insoles for ∼5 min during the familiarization period (pre‐testing) and after completing all balance tests for a given insole condition (post‐testing). After completing all balance tests for each of the insoles, participants were asked to complete a brief questionnaire, reporting their experience wearing the insoles (Supporting Information S1: Appendix 1).

### 2.4 Outcome Measures

All COP and EMG data were collected and processed using MATLAB (R2024a; The MathWorks Inc. Natick, MA, USA). Standing balance measures included peak COP path length in the anteroposterior (AP) and mediolateral (ML) directions, total COP velocity, and peak COP AP and ML range. All COP data were processed using a 10 Hz low‐pass Butterworth filter. All EMG signals were filtered with a 20–500 Hz bandpass filter, and the root‐mean‐square (RMS) was calculated over each 30 s trial. Average COP values, and EMG RMS amplitude for each muscle, were calculated across two trials per balance test. Lower limb muscle activity was normalized to the greatest value of muscle across insole conditions (within balance tests and for each muscle independently).

### 2.5 Statistical Analysis

All statistical analyses were conducted using SPSS version 29 (SPSS Inc. Chicago, IL, USA). COP, EMG, and perceived insole comfort data were not normally distributed; therefore, one‐way Friedman tests were performed to explore any differences between insole conditions for each of the four balance tests (eyes open/eyes closed × firm/foam surface) (COP, EMG), and between pre‐testing versus post‐testing (comfort). Post hoc tests were performed using pairwise comparisons with Bonferroni corrections (Dunn's test). For significant COP and EMG results, effect sizes were calculated using Cohen's *d*, and median differences and 95% confidence intervals were reported using Wilcoxon sign‐rank test. Statistical significance was set to *p* < 0.05.

## 3 Results

### 3.1 Participants

A total of 30 participants completed the study. Participant demographics and foot sensation characteristics are displayed in Table 1.

**TABLE 1:** Demographics and foot sensation characteristics for participants.

Participant demographics	Mean (SD)
Males^a^	16 (53%)
Age (years)	23.6 (3.9)
Weight (kg)	68.9 (13.8)
Height (cm)	171.1 (9.1)
Activities‐specific balance confidence scale^b^	
Total score (%)	96.0 (3.9)
Foot health status questionnaire^c^	
Foot pain	90.0 (13.2)
Foot function	96.9 (6.9)
Footwear	78.3 (23.8)
General foot health	84.1 (23.2)
General health	85.3 (17.4)
Physical activity	96.7 (9.9)
Social capacity	89.2 (14.2)
Vigor	59.8 (16.4)
Physical activity questionnaire	
Time spent doing physical activity in the last week (min)^d^	804.3 (618.3)
Number of sessions doing physical activity in the last week^e^	16.0 (6.9)
Light touch pressure sense (grams)^f^	
Great toe	0.37 (0.59)
1st metatarsal head	0.85 (0.85)
5th metatarsal head	1.11 (1.28)
Heel	1.78 (2.08)
Dorsum of foot	0.78 (0.79)
Vibration sense (Volts)^f^	
Great toe	2.93 (2.65)
1st metatarsal head	2.18 (2.30)
Ankle joint position sense (°error)^f^	2.89 (1.80)

^a^ Data are presented as the total number of males (percentage of total sample).

^b^ *Activities‐Specific Balance Confidence Scale,* subscale score range: 0%–100% (where < 50% = low level of physical functioning, 50%–80% = moderate level of physical functioning, and > 80% = high level of physical functioning).

^c^ *Foot Health Status Questionnaire,* subscale score range: 0–100 (where 0 = poorest foot health and 100 = best foot health).

Physical Activity Questionnaire:

^d^ Calculated time = walking time + moderate time + (2 × vigorous time).

^e^ Calculated number of sessions = walking sessions + moderate sessions + vigorous sessions. 0 min = sedentary, 1–149 min or 150 min or greater over < 5 sessions = insufficiently active, 150 min or greater over five or more sessions = sufficiently active for health.

^f^ *Foot sensation,* as there were no significant differences between the dominant and non‐dominant foot values within each location within every outcome (all *p* values > 0.39), the average of the dominant and non‐dominant foot was calculated.

### 3.2 Standing Balance

While standing with eyes open on a firm surface, we observed a main effect of insoles for COP ML path length (*χ* ^2^(3) = 9.480, *p* = 0.024). Post hoc analysis showed that COP ML path length was greater while wearing the vibrating insoles compared to textured insoles (*t*(29) = −2.900, *p* = 0.022, *d* = −0.53, median difference = 17.63 (95% CI: 4.58–29.23)). There were no differences between the other insole conditions (all *p* values > 0.128). There were no insole main effects for COP path length in the AP direction (*χ* ^2^(3) = 1.480, *p* = 0.687), total COP velocity (*χ* ^2^(3) = 6.360, *p* = 0.095), or COP range in the AP (*χ* ^2^(3) = 1.880, *p* = 0.598) and ML (*χ* ^2^(3) = 4.160, *p* = 0.245) direction.

Standing with eyes closed on a firm surface did not show any insole effects for COP AP path length (*χ* ^2^(3) = 2.800, *p* = 0.423), COP ML path length (*χ* ^2^(3) = 7.720, *p* = 0.052), total COP velocity (*χ* ^2^(3) = 4.200, *p* = 0.241), COP AP range (*χ* ^2^(3) = 4.040, *p* = 0.257), or COP ML range (*χ* ^2^(3) = 4.360, *p* = 0.225).

While standing with eyes open on a foam surface, there were no main effects between insole conditions for COP path length in the AP (*χ* ^2^(3) = 1.440, *p* = 0.696) and ML (*χ* ^2^(3) = 0.598, *p* = 0.598) direction. Further, there were no main effects between insoles for total COP velocity (*χ* ^2^(3) = 2.280, *p* = 0.516), COP AP range (*χ* ^2^(3) = 2.440, *p* = 0.486), or COP ML range (*χ* ^2^(3) = 2.360, *p* = 0.501).

Whilst standing with eyes closed on a foam surface, we did not observe any insole main effects for COP AP path length (*χ* ^2^(3) = 4.520, *p* = 0.211), COP ML path length: (*χ* ^2^(3) = 0.520, *p* = 0.914), total COP velocity (*χ* ^2^(3) = 1.000, *p* = 0.801), COP AP range (*χ* ^2^(3) = 3.280, *p* = 0.350), or COP ML range (*χ* ^2^(3) = 0.400, *p* = 0.940) (Table 2).

**TABLE 2:** Center of pressure measures during quiet standing balance tests whilst wearing four different insole conditions in healthy young adults (*N* = 30).

COP measure^a^	Control insole	Textured insole	Vibrating insole	Vibrotexture insole
Eyes open, firm surface				
AP path length (mm)	168.5 (146.7–214.2)	158.5 (128.7–230.5)	182.9 (140.9–223.7)	168.8 (140.2–219.7)
**ML path length (mm)**	99.5 (78.9–127.1)	96.4 (72.9–115.6)*	110.7 (77.2–147.3)*	96.3 (73.7–123.7)
Velocity (mm/s)	7.4 (6.4–9.3)	6.7 (5.5–9.6)	7.9 (6.2–9.6)	7.2 (6.0–8.7)
AP range (mm)	17.9 (16.2–24.9)	19.3 (13.6–25.6)	18.5 (15.0–26.0)	20.8 (14.8–26.4)
ML range (mm)	9.4 (6.4–13.4)	8.6 (6.8–12.8)	9.0 (6.8–15.7)	8.5 (5.8–13.9)
Eyes closed, firm surface				
AP path length (mm)	240.1 (196.1–290.2)	226.2 (177.5–292.9)	248.0 (181.6–320.6)	237.7 (188.8–324.3)
ML path length (mm)	121.9 (94.6–155.2)	107.2 (88.4–145.8)	125.7 (95.5–168.1)	108.3 (86.9–159.2)
Velocity (mm/s)	9.8 (7.6–11.9)	9.0 (7.3–13.0)	9.8 (7.5–12.5)	9.7 (7.6–12.5)
AP range (mm)	23.5 (16.5–30.4)	22.7 (19.3–30.1)	26.0 (19.3–31.8)	23.3 (19.0–34.8)
ML range (mm)	9.8 (7.1–13.7)	9.6 (6.9–13.3)	10.4 (8.2–15.4)	9.5 (7.3–12.2)
Eyes open, foam surface				
AP path length (mm)	296.7 (267.5–385.9)	304.8 (274.4–365.8)	304.5 (255.3–404.1)	316.3 (254.6–382.3)
ML path length (mm)	230.1 (193.8–290.6)	242.9 (175.2–291.4)	256.8 (174.9–319.8)	219.6 (175.3–295.6)
Velocity (mm/s)	14.7 (11.9–17.8)	14.1 (12.1–17.2)	14.9 (11.8–18.7)	14.5 (10.9–17.5)
AP range (mm)	30.5 (25.0–41.1)	33.2 (27.5–42.0)	31.0 (25.2–40.8)	29.1 (25.3–40.1)
ML range (mm)	20.3 (17.4–27.0)	21.2 (17.7–28.6)	21.5 (18.5–28.4)	23.3 (19.1–27.0)
Eyes closed, foam surface				
AP path length (mm)	668.1 (480.8–834.6)	618.1 (449.6–791.9)	645.7 (474.2–928.1)	580.6 (470.0–834.9)
ML path length (mm)	426.2 (286.5–545.1)	409.4 (311.4–533.7)	449.0 (258.3–648.4)	470.0 (274.8–554.4)
Velocity (mm/s)	28.5 (21.2–37.8)	27.9 (19.9–35.2)	30.9 (19.6–40.2)	27.9 (19.8–36.2)
AP range (mm)	52.6 (43.8–62.2)	55.9 (45.9–68.4)	56.0 (40.1–62.3)	50.4 (43.1–68.4)
ML range (mm)	35.3 (27.9–43.6)	34.2 (27.8–42.9)	35.9 (24.6–45.5)	32.3 (26.1–47.1)

*Note:* Bolded COP Measure indicates a main effect of insole.

Abbreviations: AP = anteroposterior, COP = center of pressure, ML = mediolateral.

^a^ Data are presented as median (interquartile range).

^*^ Significant difference between the two insole conditions (*p* < 0.05).

### 3.3 Lower Limb Muscle Activity

While standing with eyes open on a firm surface, we observed a significant insole effect on peroneus longus activity (*χ* ^2^(3) = 8.462, *p* = 0.037). However, post hoc analysis showed no differences between any insole conditions (all *p* values > 0.115). There was no main effect of insole conditions on the activity of medial gastrocnemius (*χ* ^2^(3) = 5.400, *p* = 0.145), soleus (*χ* ^2^(3) = 3.414, *p* = 0.332), rectus femoris (*χ* ^2^(3) = 0.422, *p* = 0.936), biceps femoris (*χ* ^2^(3) = 0.352, *p* = 0.950), or gluteus medius (*χ* ^2^(3) = 0.171, *p* = 0.982).

While standing with eyes closed on a firm surface there were no insole effects on the activity of medial gastrocnemius (*χ* ^2^(3) = 1.464, *p* = 0.691), soleus (*χ* ^2^(3) = 0.352, *p* = 0.950), peroneus longus (*χ* ^2^(3) = 2.128, *p* = 0.546), rectus femoris (*χ* ^2^(3) = 1.444, *p* = 0.695), biceps femoris (*χ* ^2^(3) = 1.345, *p* = 0.719), or gluteus medius (*χ* ^2^(3) = 1.071, *p* = 0.784).

When standing with eyes open on a foam surface, there was a significant insole effect on rectus femoris activity (*χ* ^2^(3) = 8.111, *p* = 0.044). Post hoc analysis revealed greater amplitude of rectus femoris activity while wearing the control compared to textured insoles (*t*(29) = 2.846, *p* = 0.027, *d* = 0.52, median difference = 5.6 (95% CI: −0.9 to 11.1)). There were no differences between any other insole conditions (all *p* values > 0.839). There was no main effect of insoles on the activity of medial gastrocnemius (*χ* ^2^(3) = 3.207, *p* = 0.361), soleus (*χ* ^2^(3) = 7.055, *p* = 0.070), peroneus longus (*χ* ^2^(3) = 2.048, *p* = 0.562), biceps femoris (*χ* ^2^(3) = 6.476, *p* = 0.091), or gluteus medius (*χ* ^2^(3) = 0.214, *p* = 0.975).

While standing with eyes closed on a foam surface, there were no main effects of insole condition on the activity of medial gastrocnemius (*χ* ^2^(3) = 1.841, *p* = 0.606), soleus (*χ* ^2^(3) = 7.179, *p* = 0.066), peroneus longus (*χ* ^2^(3) = 1.097, *p* = 0.778), rectus femoris (*χ* ^2^(3) = 12.378, *p* = 0.615), biceps femoris (*χ* ^2^(3) = 3.248, *p* = 0.355), or gluteus medius (*χ* ^2^(3) = 1.586, *p* = 0.663) (Table 3).

**TABLE 3:** Lower limb muscle activity during quiet standing balance tests whilst wearing four different insole conditions in healthy young adults (*N* = 30).

Muscle^a^	Control insole	Textured insole	Vibrating insole	Vibrotexture insole
Eyes open, firm surface				
Medial gastrocnemius (%)	78.1 (63.7–96.2)	83.7 (73.6–98.1)	97.6 (71.2–100.0)	78.6 (59.3–92.9)
Soleus (%)	91.4 (81.4–98.6)	97.9 (73.7–100.0)	88.8 (80.5–99.8)	88.5 (78.8–93.5)
**Peroneus longus (%)**	90.6 (83.8–95.2)	95.8 (89.6–100.0)	86.9 (69.4–96.0)	93.7 (84.4–100.0)
Rectus femoris (%)	96.1 (91.3–99.7)	95.7 (85.4–100.0)	97.8 (90.5–100.0)	96.6 (83.3–98.3)
Biceps femoris (%)	95.9 (75.3–100.0)	91.7 (73.8–99.5)	92.1 (67.0–98.0)	90.1 (70.3–98.8)
Gluteus medius (%)	95.2 (85.5–99.8)	97.7 (74.4–100.0)	94.4 (81.8–99.8)	93.6 (83.0–100.0)
Eyes closed, firm surface				
Medial gastrocnemius (%)	78.9 (68.6–94.5)	82.8 (67.2–92.6)	91.6 (71.5–100.0)	85.4 (66.8–100.0)
Soleus (%)	90.6 (72.6–100.0)	93.5 (75.9–100.0)	93.1 (82.9–98.7)	86.6 (78.8–93.6)
Peroneus longus (%)	92.5 (82.5–99.0)	93.2 (79.8–98.2)	95.9 (79.9–100.0)	88.5 (80.2–97.3)
Rectus femoris (%)	98.1 (91.1–100.0)	94.8 (88.2–99.2)	97.1 (92.5–100.0)	96.1 (81.2–98.7)
Biceps femoris (%)	87.0 (66.0–100.0)	89.1 (62.9–99.9)	92.0 (74.5–100.0)	87.6 (67.2–97.6)
Gluteus medius (%)	97.2 (82.9–100.0)	95.9 (81.8–100.0)	92.6 (81.0–97.7)	89.2 (81.2–97.9)
Eyes open, foam surface				
Medial gastrocnemius (%)	76.9 (67.7–100.0)	88.7 (72.7–98.9)	91.3 (75.9–100.0)	84.2 (63.9–92.5)
Soleus (%)	93.7 (73.8–98.4)	96.7 (88.4–100.0)	91.6 (81.3–95.6)	87.2 (79.2–97.2)
Peroneus longus (%)	87.0 (74.9–99.6)	94.5 (83.3–100.0)	83.2 (74.1–97.4)	90.0 (73.3–98.8)
**Rectus femoris (%)**	99.0 (87.9–100.0)*	91.8 (80.8–96.4)*	93.1 (81.4–97.1)	92.0 (86.8–97.3)
Biceps femoris (%)	85.6 (64.4–100.0)	88.5 (61.5–100.0)	83.1 (73.6–93.2)	73.9 (62.5–91.6)
Gluteus medius (%)	89.1 (74.2–100.0)	92.4 (79.0–100.0)	92.5 (74.9–99.2)	93.8 (79.7–98.6)
Eyes closed, foam surface				
Medial gastrocnemius (%)	72.1 (64.9–100.0)	87.1 (67.7–98.3)	74.7 (68.6–90.4)	91.1 (67.4–100.0)
Soleus (%)	88.7 (82.8–95.4)	92.1 (82.6–99.0)	92.1 (83.8–98.9)	96.1 (86.3–100.0)
Peroneus longus (%)	92.4 (76.0–100.0)	82.8 (72.9–94.7)	85.1 (71.4–95.9)	88.0 (78.9–97.9)
Rectus femoris (%)	91.6 (80.4–100.0)	93.3 (83.5–100.0)	90.0 (77.5–94.7)	87.5 (78.9–96.1)
Biceps femoris (%)	93.2 (73.2–100.0)	93.5 (62.5–100.0)	81.0 (65.5–94.8)	79.1 (54.8–86.1)
Gluteus medius (%)	91.5 (74.2–100.0)	87.7 (63.7–95.4)	91.3 (74.4–100.0)	85.4 (75.4–97.4)

*Note:* Bolded Muscle indicates a main effect of insole.

^a^ Data are presented as median (interquartile range).

^*^ Significant difference between the two insole conditions (*p* < 0.05).

### 3.4 Participant Reported Outcomes

There was a significant interaction between time and insole conditions for perceived insole comfort (*χ* ^2^(7) = 35.843, *p* < 0.001). Post hoc analysis indicated that the vibrotexture insoles were perceived to be less comfortable than the control insoles at pre‐testing (*t* (29) = 2.233, *p* = 0.012) and post‐testing (*t*(29) = 2.300, *p* = 0.008). There were no other significant differences between any time‐point or insole condition (all *p* values > 0.319) (Table 4). When participants were asked to describe how the insoles felt to wear, the most commonly reported responses included a “spiky”, “tingly”, or “rough” sensation, either across the entire foot sole, localized to the heel or toe regions, or the foot region was not specified (Table 5).

**TABLE 4:** Perceived level of insole comfort whilst wearing four different insole conditions in healthy young adults^a^.

	Control insole	Textured insole	Vibrating insole	Vibrotexture insole
Pre‐testing	90.0 (70.0–100.0)*	80.0 (68.8–90.0)	80.0 (71.5–90.0)	80.0 (60.0–90.0)*
Post‐testing	87.5 (73.8–95.0)*	80.0 (67.5–90.0)	80.0 (65.0–90.0)	77.5 (59.3–90.0)*

^a^ Data are presented as median (interquartile range).

^*^ Significant difference between the two insole conditions (*p* < 0.05).

**TABLE 5:** Occurrence of the most commonly reported responses of how the insoles felt to wear, across time and insole conditions at each region of the foot.

Response		Location on plantar surface			
Insole	Time^a^	Sole	Heel	Toes	Not specified
“Spiky”	Pre‐testing (*N* = 6)	3	3	2	0
Textured	Post‐testing (*N* = 3)	1	1	0	1
Vibrotexture	Pre‐testing (*N* = 2)	0	0	1	1
	Post‐testing (*N* = 2)	0	0	0	2
“Tingly”					
Textured	Pre‐testing (*N* = 1)	1	0	0	0
	Post‐testing (*N* = 1)	1	0	0	0
Vibrating	Pre‐testing (*N* = 2)	0	1	0	1
	Post‐testing (*N* = 1)	1	0	0	0
Vibrotexture	Pre‐testing (*N* = 2)	2	0	0	0
	Post‐testing (*N* = 5)	1	1	2	1
“Rough”					
Textured	Pre‐testing (*N* = 4)	1	0	3	0
Vibrotexture	Pre‐testing (*N* = 3)	2	0	1	0

^a^ *N* = number of participants who reported this outcome at each time point.

## 4 Discussion

The aim of this study was to explore the immediate effects of wearing vibrotexture insoles on measures of standing balance and lower limb muscle activity in healthy young adults, compared to textured insoles, vibrating insoles, and control insoles. Although textured and vibrating insoles have, separately, lead to immediate changes in quiet standing balance (e.g., reduced COP measures) in healthy young adults, this study is the first to combine these two types of stimuli into a hybrid insole design. Contrary to the hypothesis, vibrotexture insoles delivering multiple‐sensory stimuli did not appear to bring about any greater benefits on balance control over single‐sensory stimulus insole design. However, when wearing textured insoles, a significant reduction in COP ML path length was observed compared to vibrating insoles while standing with eyes open on a firm surface. In addition, wearing textured insoles also led to a reduction in the amplitude of rectus femoris activity compared to control insoles while standing with eyes open on a foam surface.

The foot sensation characteristics of the participants suggest that they were fully sensate (Table 1). Consequently, in populations with full foot sensation, it is possible that sensory‐stimulating insoles may provide an overload of sensory information for balance control. In healthy athletes, such as ballet dancers, textured insoles did not improve static balance [25]. The authors proposed that given the female dancers had full sensation on their feet, the additional sensory input from the textured insoles may have been redundant [26]. Further, Corbin et al. [27] demonstrated that, during single‐leg stance, textured insoles did not provide benefits for balance control in healthy individuals, suggesting an excess of sensory information. The vibrotexture insoles used in the present study, with multiple‐sensory stimuli, may have delivered an overload of plantar surface stimulation for the quiet standing balance task, which may explain why there were no improvements compared to the other insoles.

It is also possible that the quiet standing balance task was not challenging enough to bring about any additional benefits of vibrotexture insoles over single‐sensory stimulus insole designs in healthy young adults. More dynamic balance tasks, such as responding to a perturbation [28], standing in a more challenging posture [29], or walking over uneven terrain [3] may provide a greater threat to the balance system and the potential effects of vibrotexture insoles may become more apparent. Steinberg et al. [29] investigated the effects of textured insoles and surfaces on balance control in ballet dancers performing single‐leg flexion–extension in a ballet posture. The enhanced plantar surface stimulation appeared to provide beneficial effects on balance control, suggesting that the dancers may have relied more heavily on the enhanced sensory input provided by the textured insoles or surface to maintain their balance. However, as the balance task used by Steinberg et al. [29] does not directly reflect a typical activity of daily living, future studies should examine vibrotexture insoles during perturbations, or walking or running over uneven terrain.

A reduction in COP ML path length was observed while wearing the textured insoles compared to vibrating insoles while standing with eyes open on a firm surface. Lower COP values typically indicate improved postural control [30], and ML changes are particularly relevant given their association with fall risk [31, 32]. Consistent with Hatton et al. [16], which reported reduced ML sway on a textured surface in healthy older adults, our findings suggest textured insoles may beneficially influence ML sway characteristics linked to reduced falls, and provide a mechanistic basis for future studies in older and clinical populations. However, the effects of textured insoles on COP measures during quiet stance are generally reported to be more pronounced when standing with eyes closed [6, 27, 33] or on a compliant surface [7], likely due to sensory reweighting that may rely more heavily on other sources of sensory input for balance control [14], such as the cues from the textured insoles. Therefore, the observation of a significant difference between insole conditions during a balance test where all sensory input is present (i.e., eyes open, firm surface) was contrary to expectations. Further, it is important to note that the finding of reduced COP ML path length represents only one significant finding from a total of 20 COP measures and should be interpreted with caution.

A reduction in the amplitude of rectus femoris activity occurred while wearing textured insoles compared to control insoles, while standing with eyes open on a foam surface. Although no simultaneous improvements in COP measures occurred, it is possible that the textured insoles still provided a more energy‐efficient postural strategy during quiet stance by reducing muscle activity [34]. This change in neuromuscular control may not translate into measurable changes in sway during quiet standing, but it highlights a subtle benefit in reducing lower limb muscle activation. Such efficiency gains could become more critical in more demanding balance tasks or in populations where muscle fatigue and reduced foot sensation compromise postural stability (e.g., peripheral neuropathy due to aging, diabetes, etc.).

The vibrotexture insoles were rated as less comfortable than the control insoles (Table 4), consistent with previous reports on sensory‐stimulating insoles [3, 35]. Participants commonly described the insoles as “spiky,” “tingly,” or “rough,” (Table 5), suggesting that future designs may benefit from enhancing comfort, possibly by reducing sharpness of the texture (e.g., rounder or softer elements) or moderating vibration intensity, while maintaining the intended sensory stimulation.

### 4.1 Limitations

This study has several limitations. First, participants wore the insoles only during the laboratory assessments. However, the benefits of sensory‐stimulating insoles may accumulate over time with prolonged wear in the community. Second, benefits from sensory‐stimulating insoles may become more evident on other aspects of balance (e.g., anticipatory or reactive) or neuromuscular (e.g., spinal excitability) control that were not assessed in the current study. Finally, the vibrating insoles delivered intermittent perceptible vibration under the heel and forefoot that was synchronized with the direction of sway. In addition to enhanced sensory information, it is possible that the vibratory cues may have also provided participants with real‐time biofeedback to modify their balance, warranting further investigation in future research.

## 5 Conclusion

Wearing vibrotexture insoles for the first time did not alter measures of quiet standing balance or lower limb muscle activity in healthy young adults. However, we provide evidence that textured insoles can reduce ML path length and rectus femoris activity. Further investigation of multi‐sensory stimuli insole design should focus on balance and gait tasks with greater complexity, and populations with diminished foot sensation (e.g., peripheral neuropathy), where the insoles may have a greater therapeutic effect.

## Author Contributions

**Megan Trotman:** conceptualization, data curation, formal analysis, investigation, methodology, project administration, software, validation, visualization, writing – original draft, writing – review and editing. **Avni Hurkat:** formal analysis, investigation, project administration, visualization, writing – review and editing. **Kylie Tucker:** conceptualization, data curation, formal analysis, investigation, methodology, project administration, resources, software, supervision, validation, visualization, writing – review and editing. **Thomas Cattagni:** conceptualization, data curation, formal analysis, investigation, methodology, supervision, validation, writing – review and editing. **Anna L. Hatton:** conceptualization, data curation, formal analysis, investigation, methodology, project administration, resources, software, supervision, validation, visualization, writing – review and editing.

## Funding

The authors have nothing to report.

## Conflicts of Interest

The vibrating insoles used in this study were provided by Walk With Path (London/Copenhagen). Walk With Path was not involved in any aspects of study design, analysis or interpretation of findings.

## Supporting information


Supporting Information S1

## Data Availability

The data that support the findings of this study are available from the corresponding author upon reasonable request.
