# Supplementary material for: The Immediate Effects of Vibrotexture Shoe Insoles on Quiet Standing Balance and Lower Limb Muscle Activity in Healthy Young Adults
Source: J Foot Ankle Res. 2026 Mar 19;19(1):e70150. doi: 10.1002/jfa2.70150 (PMC13097555; doi:10.1002/jfa2.70150)
Supplement: Supplementary file 1 — Supporting Information S1 [file JFA2-19-e70150-s001.pdf]

Perception Measures

Participant #__________

Control/Smooth Insole – Initial Use

Date:____________________

1. Comfort level

With regard to the current insole that you are wearing, please rate your level of comfort by placing a single vertical line on the scale

Overall Comfort	
Extremely uncomfortable	Extremely comfortable

2. Changes in Sensation:

i) Please describe any changes in sensation in your feet.
ii) At what region(s) of your feet are you experiencing these changes?

Control/Smooth Insole – End of Use

1. Comfort level

With regard to the current insole that you are wearing, please rate your level of comfort by placing a single vertical line on the scale

Overall Comfort	
Extremely uncomfortable	Extremely comfortable

2. Changes in Sensation:

iii) Please describe any changes in sensation in your feet.
iv) At what region(s) of your feet are you experiencing these changes?

Perception Measures

Participant #__________

Textured Insole – Initial Use

Date:___________________

1. **Comfort level**

With regard to the current insole that you are wearing, please rate your level of comfort by placing a single vertical line on the scale

Overall Comfort	
____	
Extremely uncomfortable	Extremely comfortable

2. **Changes in Sensation:**

v) Please describe any changes in sensation in your feet.
vi) At what region(s) of your feet are you experiencing these changes?

____

____

Textured Insole – End of Use

1. **Comfort level**

With regard to the current insole that you are wearing, please rate your level of comfort by placing a single vertical line on the scale

Overall Comfort	
____	
Extremely uncomfortable	Extremely comfortable

2. **Changes in Sensation:**

vii) Please describe any changes in sensation in your feet.
viii) At what region(s) of your feet are you experiencing these changes?

____

____

Perception Measures

Participant #__________

Vibrating Insole – Initial Use

Date:___________________

1. Comfort level

With regard to the current insole that you are wearing, please rate your level of comfort by placing a single vertical line on the scale

Overall Comfort	
____	
Extremely uncomfortable	Extremely comfortable

2. Changes in Sensation:

ix) Please describe any changes in sensation in your feet.
x) At what region(s) of your feet are you experiencing these changes?

____

____

Vibrating Insole – End of Use

1. Comfort level

With regard to the current insole that you are wearing, please rate your level of comfort by placing a single vertical line on the scale

Overall Comfort	
____	
Extremely uncomfortable	Extremely comfortable

2. Changes in Sensation:

xi) Please describe any changes in sensation in your feet.
xii) At what region(s) of your feet are you experiencing these changes?

____

____

Perception Measures

Participant #__________

Vibrotexture Insole – Initial Use

Date:____________________

1. Comfort level

With regard to the current insole that you are wearing, please rate your level of comfort by placing a single vertical line on the scale

Overall Comfort	
Extremely uncomfortable	Extremely comfortable

2. Changes in Sensation:

xiii) Please describe any changes in sensation in your feet.

xiv) At what region(s) of your feet are you experiencing these changes?

____

____

Vibrotexture Insole – End of Use

1. Comfort level

With regard to the current insole that you are wearing, please rate your level of comfort by placing a single vertical line on the scale

Overall Comfort	
Extremely uncomfortable	Extremely comfortable

2. Changes in Sensation:

xv) Please describe any changes in sensation in your feet.

xvi) At what region(s) of your feet are you experiencing these changes?

____

____
